# Isoform-specific promotion of breast cancer tumorigenicity by TBX3 involves induction of angiogenesis

**DOI:** 10.1038/s41374-019-0326-6

**Published:** 2019-09-30

**Authors:** Milica Krstic, Haider M. Hassan, Bart Kolendowski, M. Nicole Hague, Pieter. H. Anborgh, Carl O. Postenka, Joseph Torchia, Ann F. Chambers, Alan B. Tuck

**Affiliations:** 1 Department of Oncology, Schulich School of Medicine and Dentistry, Western University, London, ON Canada; 2 The Pamela Greenaway-Kohlmeier Translational Breast Cancer Research Unit, London Regional Cancer Program, London Health Sciences Centre, London, ON Canada; 3 Department of Pathology, Schulich School of Medicine and Dentistry, Western University, London, ON Canada; 4 Department of Biochemistry, Schulich School of Medicine and Dentistry, Western University, London, ON Canada; 5 Mary & John Knight Translational Ovarian Cancer Research Unit, London Regional Cancer Program, London Health Sciences Centre, London, ON Canada

**Keywords:** Breast cancer, Oncogenes, Tumour angiogenesis, Mechanisms of disease

## Abstract

TBX3 is a member of the highly conserved family of T-box transcription factors involved in embryogenesis, organogenesis and tumor progression. While the functional role of TBX3 in tumorigenesis has been widely studied, less is known about the specific functions of the different isoforms (TBX3iso1 and TBX3iso2) which differ in their DNA-binding domain. We therefore sought to investigate the functional consequence of this highly conserved splice event as it relates to TBX3-induced tumorigenesis. By utilizing a nude mouse xenograft model, we have identified differential tumorigenic potential between TBX3 isoforms, with TBX3iso1 overexpression more commonly associated with invasive carcinoma and high tumor vascularity. Transcriptional analysis of signaling pathways altered by TBX3iso1 and TBX3iso2 overexpression revealed significant differences in angiogenesis-related genes. Importantly, osteopontin (OPN), a cancer-associated secreted phosphoprotein, was significantly up-regulated with TBX3iso1 (but not TBX3iso2) overexpression. This pattern was observed across three non/weakly-tumorigenic breast cancer cell lines (21PT, 21NT, and MCF7). Up-regulation of OPN in TBX3iso1 overexpressing cells was associated with induction of hyaluronan synthase 2 (HAS2) expression and increased retention of hyaluronan in pericellular matrices. These transcriptional changes were accompanied by the ability to induce endothelial cell vascular channel formation by conditioned media in vitro, which could be inhibited through addition of an OPN neutralizing antibody. Within the TCGA breast cancer cohort, we identified an 8.1-fold higher TBX3iso1 to TBX3iso2 transcript ratio in tumors relative to control, and this ratio was positively associated with high-tumor grade and an aggressive molecular subtype. Collectively, the described changes involving TBX3iso1-dependent promotion of angiogenesis may thus serve as an adaptive mechanism within breast cancer cells, potentially explaining differences in tumor formation rates between TBX3 isoforms in vivo. This study is the first of its kind to report significant functional differences between the two TBX3 isoforms, both in vitro and in vivo.

## Abstract (web summary)

While the transcription factor TBX3 is widely studied in tumorigenesis, the functionality of the two isoforms (TBX3iso1 and TBX3iso2) which differ in their DNA-binding domain is not well understood. By utilizing a nude mouse xenograft model, the authors identified differential tumorigenic potential between TBX3 isoforms, with TBX3iso1 (but not TBX3iso2) promoting invasive carcinoma in vivo through induction of angiogenesis. This process is moderated by downstream mediators osteopontin (OPN) and hyaluronan synthase 2 (HAS2).

## Introduction

TBX3 is a member of the highly conserved family of T-box transcription factors involved in embryogenesis and organogenesis. Germline mutations or haploinsufficiency of TBX3 results in ulnar-mammary syndrome (UMS, OMIM 181450), characterized by mammary gland hypoplasia, apocrine gland, dental, and genital defects, emphasizing its broad expression profile [1, 2]. TBX3 levels are additionally up-regulated in several cancer types, including breast [3–6], melanoma [7], colorectal [8], pancreatic [9], cervical [10], ovarian [4], gastric [11], and prostate cancers [12], suggesting its potential role as an oncogenic driver in multiple cancer types.

Two predominant isoforms of TBX3 are produced through alternative splicing: TBX3iso1 and TBX3iso2. TBX3iso2 contains a unique 20 amino acid sequence (attributed to the 2a exon) which is inserted into the DNA-binding domain [2]. Addition of the 2a exon within TBX3iso2 shifts residues critical for interaction with target DNA sequences known as T-box elements (TBEs) [13]. Furthermore, the DNA-binding domain which differs between the two TBX3 isoforms facilitates several protein–protein interactions, including those with core histones and chromatin-interacting proteins such as methyltransferases [14–16]. The functional consequence of this highly conserved splice event, however, remains unclear. Fan et al., reported that the 20 amino acid addition in TBX3iso2 hinders its binding to a previously identified TBE, as assessed through in vitro oligonucleotide binding assays, suggesting that the protein’s DNA binding is altered [3]. In a study by Hoogaars et al., both TBX3 isoforms were able to bind to the TBE in the *Nppa* and *p21*^*CIP1*^ promoters in vitro, suggesting that they may have similar functions with regards to these promoters [17]. Due to the growing literature implicating TBX3 in the promotion of tumorigenesis in several cancer types [3–7, 9, 10, 12, 18, 19], and the observation of tissue- and species-specific TBX3 isoform ratios [3], it is essential to address the functional relevance of TBX3 isoforms and their altered expression in cancer.

In order to characterize the roles of TBX3 in tumorigenesis in an isoform-specific context, we conducted in vivo nude mouse xenograft experiments. Importantly, we observed differential tumorigenicity between TBX3 isoforms when expressed in non-tumorigenic 21NT breast epithelial cells, with TBX3iso1 associated with invasive carcinoma and high tumor vascularity. High throughput RNA sequencing and subsequent pathway analysis revealed an enrichment of angiogenesis-promoting transcripts in tumorigenic TBX3iso1 overexpressing cells. Downstream functional characterization showed enhanced promotion of angiogenesis by TBX3iso1, with osteopontin (OPN) acting as a critical downstream mediator in this process. This phenotype was not observed in TBX3iso2 overexpressing cells. This study is the first of its kind to report significant functional differences between the two TBX3 isoforms, both in vitro and in vivo, beginning with TBX3iso1-dependent promotion of angiogenesis and resulting in tumor initiation and progression.

## Methods

### Cell lines and culture conditions

The 21T series cell lines (21PT and 21NT) were obtained as a gift from Dr Vimla Band (Dana Farber Cancer Institute) [20]. The 21PT and 21NT cell lines and transfectants underwent cell line authentication by Idexx Radil (IDEXX Bioanalytics, Missouri, CA, USA; Case No. 20250-2013). The 21PT and 21NT cell lines were maintained in alpha modification of minimum essential medium (αMEM) supplemented with 2 mM l-glutamine, 1 µg/mL insulin, 12.5 ng/mL epidermal growth factor (EGF), 2.8 µM hydrocortisone, 10 mM hydroxyethyl-piperazineethane-sulfonic acid buffer (HEPES), 1 mM sodium pyruvate, 0.1 mM nonessential amino acids, 50 µg/mL gentamycin sulfate, and 10% fetal bovine serum (FBS). MCF7 cells were obtained from ATCC (Manassas, VA, USA) and maintained in Dulbecco’s modified eagle medium (DMEM) supplemented with 10% FBS. Stable 21PT, 21NT, and MCF7 transfectants were maintained in their respective media supplemented with 500 µg/mL Geneticin (G418) as a selection marker. All reagents for culture of breast cancer (BRCA) cell lines were obtained from Wisent Inc (Saint-Jean-Baptiste, QC, Canada). Human neonatal dermal microvascular endothelial cells (HDMECs) were obtained from Lonza (Basel, Switzerland; CC2516). Cells were expanded in endothelial basal media-2 (EBM-2; Lonza, 00190860) supplemented with 20% FBS and SingleQuots (Lonza, CC-4176) growth factors. The minimum density for subculturing was maintained at 2500 cells/cm^2^. Only HDMECs under passage 10 were used for experiments.

### Generation of stable transfectant cell lines

Stable transfectants were generated using plasmid constructs previously described [18], consisting of either an empty vector (EV), TBX3iso1, or TBX3iso2 construct within a pcDNA3.1 vector (Invitrogen, Carlsbad, CA, USA; V79020). Briefly, cells (21PT, 21NT, and MCF7) were seeded into six-well plates at 350,000 cells per well. The following day, cells were transfected using the Lipofectamine 3000 Transfection Kit (Invitrogen, L3000; 3 µg of plasmid DNA per well) as per the manufacturer’s protocol. Selection was performed using the aforementioned media for each cell line, further supplemented with 500 µg/mL G418. Resistant clones were pooled, expanded, and frozen for later use.

### RNA isolation and quantitative real-time PCR (qRT-PCR)

RNA was isolated using the RNeasy Mini Kit (Qiagen, Venlo, Netherlands; 74104) and converted into cDNA using the qScript cDNA SuperMix (Quanta Biosciences, Beverly, MA, USA; 84034). The RT^2^ SYBR Green ROX qRT-PCR Mastermix (Qiagen, 330521) was utilized for quantitative PCR, with the primer sequences listed in Table 1. The output values were normalized to GAPDH expression using the ∆∆Ct method, and are shown as fold changes relative to the empty vector control. Means derived from a minimum of three biological replicates were used during analysis.

**Table 1:** Primer sequences utilized for qRT-PCR in mRNA studies

mRNA probe	Primer sequences (forward and reverse, 5′ to 3′)
Total TBX3	F: CGCTGTGACTGCATACCAGA R: GTGTCCCGGAAACCTTTTGC
TBX3iso1	F: AGTGGATGTCCAAAGTCGTCAC R: CATGGAGTTCAATATAGTAAATCCATGTTTGTCTG
TBX3iso2	F: AGTGGATGTCCAAAGTCGTCAC R: CACTTGGGAAGGCCAAAGTAAATCCATG
GAPDH	F: AGGCTGGGGCTCATTTGAAG R: CCATCCACAGTCTTCTGGGTG
COX1	F: CGCCAGTGAATCCCTGTTGT R: GTCACACTGGTAGCGGTCAA
HAS2	F: GTTGGGGGAGATGTCCAGATTT R: CGGTTCGTGAGATGCCTGT
IL1RN	F: AGCAAGATGCAAGCCTTCAG R: CCTTGCAAGTATCCAGCAACTA
OPN	F: TTGCAGTGATTTGCTTTTGC R: TCAATGGAGTCCTGGCTGTC
VEGFR2	F: CCCAGATGACAACCAGACGG R: GCCTTCAGATGCCACAGACT

### Nude mouse xenografts

Cells were grown to confluence on 150 mm tissue culture dishes. Cells were harvested, washed twice with ice cold phosphate buffered saline (PBS), and resuspended in serum-free αHE media. The cell suspension (100 µL containing 1.0 × 10^7^ cells) was injected into the second thoracic mammary fat pad of 8–9-week-old female nude mice at ten mice per group (one mouse in the TBX3iso2 group developed lymphoma and was removed from the study). Mice were monitored regularly for tumor growth up to 1 year post injection and euthanized either when tumors reached a volume of 2500 mm^3^ or 1 year post injection, whichever occurred first. The tumor volume was calculated using the formula: V = π/6(L × W^2^), where V = volume, L = length (longer dimension), and W = width (smaller dimension) as measured by digital calipers. The primary tumor and/or tumor-free mammary fat pad, along with brain, liver, spleen, kidneys, lungs, and lymph nodes (axillary, brachial, and inguinal) were collected. All tissues were formalin-fixed, paraffin-embedded, sectioned, and H&E stained. Animal care and surgical procedures were conducted in accordance with the recommendations of the Canadian Council on Animal Care, under a protocol approved by Western University’s Council on Animal Care.

### Immunohistochemistry of mouse xenograft tissues

Formalin-fixed, paraffin-embedded tissues were sectioned at 4.0 µm thickness onto charged glass slides. Sections were deparaffinized and rehydrated. Antigen retrieval was conducted with 10 mM citrate buffer (pH 6.0) for 20 min, maintaining sub-boiling conditions. The UltraVision LP Detection System (Thermo Fischer Scientific, Waltham, MA, USA; TL-015-HD) was used as per the manufacturer’s protocol. Suspect tissues and lymph nodes were stained using a mouse anti-human mitochondrial antibody (Thermo Fischer Scientific, MS-1372-P0; 1/100 for 20 min at room temperature) to confirm metastases [21]. The degree of angiogenesis across primary tumors was assessed using a rabbit anti-mouse CD31 antibody (Abcam, Cambridge, UK; ab28364; 1/50 at 4 °C overnight). OPN expression across primary tumors was assessed using a mouse anti-human OPN mAb53 antibody (Enzo Life Sciences, Farmingdale, NY, USA; ADI-905-629; 1/750 for 90 min at room temperature). Signal for all stains was developed using 3,3′Diaminobenzidine (DAB), and slides were counter-stained in Harris’s Hematoxylin. Expression of human mitochondria was classified as either positive or negative. For quantification of CD31 expression by immunohistochemistry, images of ten non-overlapping areas exhibiting high vessel density were acquired using an Olympus IX70 inverted microscope with a ×10 objective, and the number of vessels per high-power field was assessed using ImageJ. For quantification of OPN expression by immunohistochemistry, images of ten random, non-overlapping areas were acquired using the Olympus IX70 inverted microscope with a ×10 objective. Images were imported to ImageJ, and color deconvolution using the H DAB setting was conducted. DAB images were thresholded, and the percentage of positive signal within the image area was quantified.

### In vitro endothelial tubule formation assay

In vitro endothelial tubule formation assays were conducted as previously described [22]. Briefly, HDMECs were grown to 80% confluency. Growth factor reduced Matrigel (Thermo Fischer Scientific, CB356239) was added to 96-well plates (50 µL/well). HDMECs were trypsinized, washed twice with PBS, and resuspended at 2.0 × 10^5^ cells/mL in media (1:1 mixture of conditioned media and basal EBM-2 media with 20% FBS and no SingleQuots growth factors). Conditioned media was prepared by seeding 1.0 × 10^6^ cells into T75 flasks, and cells were maintained in low-serum media (αMEM with 0.1% FBS) for 48 h. The endothelial cells suspended in conditioned media were added to the 96-well plate on top of the Matrigel (100 µL, 20,000 cells per well) and incubated at 37 °C for 16 h. For OPN neutralization experiments, anti-OPN mAb53 antibody (Enzo Life Sciences, ADI-905-629) was added to the conditioned media and cell suspension at a concentration of 20 µg/mL prior to plating. For all studies, tubule formation was observed after 16 h, and images were taken of three non-overlapping fields of view per well using the Olympus IX70 inverted microscope with a ×10 objective. The number of endothelial cell tubules formed per well were counted using ImageJ (represented as branch points per three high-power fields).

### Analysis of secreted OPN by ELISA

Cells were seeded at a density of 1.0 × 10^6^ into T75 flasks (with 10 mL media) and grown in serum-free αMEM media (with the aforementioned supplements) for 48 h. Conditioned media (100 µL) were subject to ELISA using a Dual Mono ELISA kit (Enzo Life Sciences, Farmingdale, NY, USA; ADI-900-142) as previously described [23, 24]. Human recombinant OPN provided within the kit was used to create a standard curve in order to determine absolute concentrations. OPN levels were normalized to equal amounts of cells.

### Particle exclusion assay

Particle exclusion assays were conducted to visualize pericellular matrices, as previously described [25]. Briefly, 15,000 cells were plated in six-well plates in triplicate and allowed to adhere overnight. The following day, cells were pretreated in the presence or absence of hyaluronidase (HAse) (Sigma, St. Louis, MO, USA; H1136; 16 U/mL in αHE media with 0.1% BSA) for 20 min at 37 °C. The media was then removed, and fixed sheep erythrocytes (Innovative Research, Novi, MI, USA; IC100-0210) were added and allowed to settle for 10 min. Plates were imaged using the phase contrast setting on an Olympus IX70 inverted microscope at ×10 objective. Pericellular matrices appeared as halos surrounding cell surfaces from which erythrocytes were excluded. In order to quantify matrix production, a ratio of the pericellular matrix area over the cell area was calculated by tracing around the cell coats (matrices) and cell areas of 30 randomly selected cells using ImageJ. A ratio of 1.0 indicated the absence of a pericellular matrix for a particular cell.

### Bioinformatics analyses

RNA-Seq data from 21NT + EV, 21NT + TBX3iso1, and 21NT + TBX3iso2 cell lines previously published by our group [19] was used to examine differences in resultant transcriptional profiles with overexpression of TBX3iso1 and TBX3iso2 (GEO accession number: GSE126153). Assessment of differentially expressed genes was conducted by compiling a list of genes significantly up or down-regulated (>1.5-fold, FDR < 0.05) in only one isoform relative to the empty vector control, or showed opposite patterns between isoforms (>1.5-fold up in one, >1.5-fold down in the other, all FDR < 0.05). Enrichment analysis (Enrichr; Icahn School of Medicine, Mount Sinai; http://amp.pharm.mssm.edu/Enrichr/) was then conducted for cells overexpressing either TBX3iso1 or TBX3iso2 relative to the empty vector control, focusing on genes altered >1.5-fold up or down for only one isoform, or >1.5-fold in opposite directions for both isoforms, and all with corrected FDR < 0.05 (http://amp.pharm.mssm.edu/Enrichr/). Results from WikiPathways analysis are reported. The combined score takes into account the *p* value and *z* value, with the calculation Combined Score = ln(*p*)*z, with *p* representing the *p* value and *z* representing the *z*-score [26].

An angiogenesis gene signature consisting of 222 genes was compiled by integration of genes with the Ingenuity Pathway Analysis (Qiagen) gene ontology term “angiogenesis of tumor”, along with the angiogenesis gene lists from the databases in Table 2 (top four datasets).

**Table 2:** Publicly-available datasets utilized for analysis

Project	Dataset	Reference (or link)
Angiogenesis	MsigDB (Broad Institute)	http://software.broadinstitute.org/gsea/index.jsp
Angiogenesis	PubAngioGen (East China Normal University)	http://www.megabionet.org/aspd Li et al. [76]
Angiogenesis	AngioDB (Pusan National University)	Sohn et al. [77]
Angiogenesis	dbANGIO (Memorial University of Newfoundland)	http://www.med.mun.ca/angio/ Savas et al. [78]
Isoform Analysis	ICGC (US Donors)	https://xenabrowser.net/datapages/
Isoform Analysis	TCGA breast cancer (BRCA)	https://portal.gdc.cancer.gov/projects/TCGA-BRCA
Isoform Analysis	TCGA BRCA grade information	Budczies et al. [27]
Isoform Analysis	GTEx (Genotype Tissue Expression)	https://xenabrowser.net/transcripts/

The International Cancer Genome Consortium (ICGC, US donors) data was exported using XenaBrowser (University of California, Santa Cruz; https://xenabrowser.net/datapages/), and total TBX3 transcript levels were assessed across all cancer subtypes. Transcript levels of TBX3 isoforms were acquired from the TCGA portal, and examined in the BRCA dataset. A ratio of total transcript reads for TBX3iso1 (uc001tvu) over total transcript reads for TBX3iso2 (uc001tvt) was compared against clinical data and tumor characteristics. Grade information for patients within the TCGA BRCA was obtained from Budczies et al. [27]. The TCGA and GTEx datasets were compared simultaneously through the use of the “transcripts” function in XenaBrowser. The aforementioned datasets are listed in Table 2 (bottom four datasets).

### Statistical analysis

Statistical analyses were conducted using GraphPad Prism 8. One-way ANOVA with Tukey post-hoc tests were conducted for the majority of analyses unless otherwise specified. Error bars are representative of standard deviation measurements. *P*-values less than 0.05 were considered statistically significant.

## Results

### TBX3iso1 possesses enhanced tumorigenic potential in nude mice

**Fig. 1:**
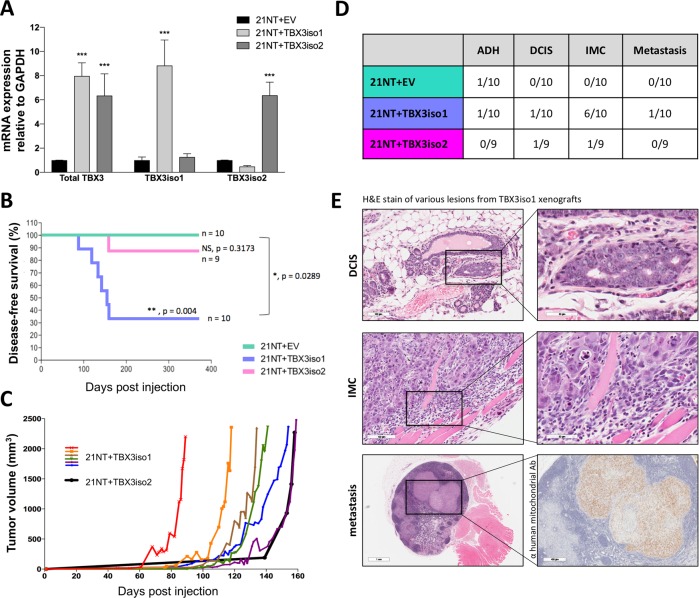
TBX3iso1 possess enhanced tumorigenic potential in nude mice. **a** Total TBX3, TBX3iso1, and TBX3iso2 mRNA expression was assessed by qRT-PCR, normalized to GAPDH expression levels, and depicted as fold change relative to the empty vector control. **b** Cells (1.0 × 10^7^) were injected into the mammary fat pad of nude mice. Mice were sacrificed when the tumor volume reached 2500 mm^3^, or 1-year post injection, whichever occurred first. The Kaplan–Meier plot shows disease-free survival over 365 days post injection. An event was defined as tumor volume reaching the 2500 mm^3^ end-point. **c** Tumor volume showing growth kinetics over time. TBX3iso1 tumors are shown in various colors, while the TBX3iso2 tumor is shown in black. **d** Histological analysis of H&E stained slides was conducted by an anatomical pathologist for all mammary fat pads and primary tumors. Cases with atypical ductal hyperplasia (ADH), ductal carcinoma in situ (DCIS), invasive mammary carcinoma (IMC), and metastasis were documented. All tissues and lymph nodes collected were examined for metastases in mice with IMC. Metastases were confirmed through positive immunohistochemical staining for anti-human mitochondria. **e** Representative images of cases of DCIS, IMC showing invasion into skeletal muscle, and metastasis into the right brachial lymph node. Suspect metastases from H&E slides were confirmed through immunohistochemical staining with anti-human mitochondrial antibody. *p < 0.05, **p < 0.01, ***p < 0.001 by one-way ANOVA with Tukey post-hoc for comparison between three groups. Survival analysis for Kaplan–Meier curve was calculated using the log-rank test statistic. Error bars represent standard deviation

To examine the tumorigenicity of TBX3 isoforms, we stably overexpressed either TBX3iso1 or TBX3iso2 in non-tumorigenic, ductal carcinoma in situ (DCIS)-like 21NT breast cancer  cell lines (Fig. 1a). Cells were injected into the mammary fat pad of nude mice, and mice were monitored for up to 1 year for tumor growth. The majority of mice (6/10) injected with cells overexpressing TBX3iso1 developed invasive carcinoma and reached the endpoint tumor volume, a rate significantly higher than mice injected with cells overexpressing TBX3iso2 (1/9; *p* < 0.05 relative to TBX3iso1) or the empty vector control (0/10; *p* < 0.01 relative to TBX3iso1) (Fig. 1b–d). In addition, the TBX3iso1 tumors exhibited a shorter lag period of in vivo growth, all forming before the single TBX3iso2 tumor (Fig. 1c). All primary tumors (or apparently tumor-free mammary fat pad injection site at gross examination) were assessed histologically for the presence of precursor lesions (atypical ductal hyperplasia, ADH; DCIS) (Fig. 1d). Representative images of histological lesions observed are shown in Fig. 1e, representing DCIS, invasive mammary carcinoma (IMC) invading into skeletal muscle, and a metastatic lesion within the brachial lymph node (Fig. 1e). All collected organs were assessed for metastases, and suspect tissues were stained using an anti-human mitochondrial antibody for confirmation (Fig. 1e, bottom right panel).

### TBX3iso1 promotes angiogenesis in vivo and in vitro

**Fig. 2:**
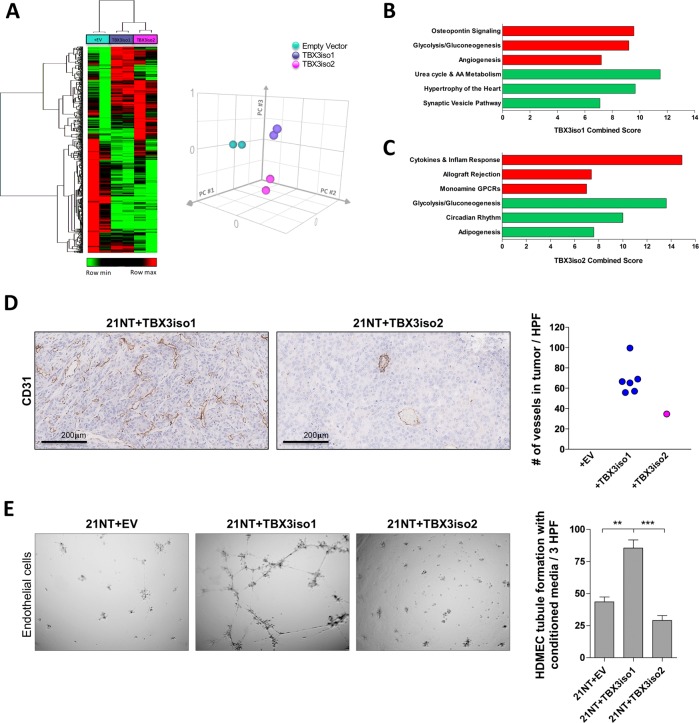
TBX3iso1 promotes angiogenesis in vitro and in vivo. **a** Heat map showing large-scale transcriptional changes by RNA-Seq across 21NT + EV, 21NT + TBX3iso1, and 21NT + TBX3iso2 cell lines (left panel). Principle component analysis (PCA) was conducted to assess the similarity in global transcriptional profiles between transfectant cell lines (right panel). **b** Enrichment analysis highlighting the top pathways associated with transcripts up-regulated (red) and down-regulated (green) with TBX3iso1 overexpression relative to the empty vector control. **c** Enrichment analysis highlighting the top pathways associated with transcripts up-regulated (red) and down-regulated (green) with TBX3iso2 overexpression relative to the empty vector control. **d** Assessment of microvascular density within primary tumors. Primary tumors were stained for CD31 by immunohistochemistry and the number of vessels per non-overlapping high power field was quantified across ten microvascular hotspots. Averages across ten hotspots for each mouse are shown. **e** Tubule formation assay to asses in vitro angiogenesis. Conditioned media was collected after a 48 h incubation with 1.0 × 10^6^ cells of each cell type. Conditioned media was incubated with human dermal microvascular endothelial cells (HDMEC) on growth factor reduced Matrigel for 16 h at 37 °C to allow for vascular channel formation. The number of tubule branch points per three high-power fields (one well) was quantified at the 16 h mark. *p < 0.05, **p < 0.01, ***p < 0.001 by one-way ANOVA with Tukey post-hoc for comparison between three groups. Error bars represent standard deviation

The differential tumorigenicity between TBX3 isoforms was a striking and unanticipated observation. In order to assess transcriptional differences that may explain these novel findings, we re-examined our RNA-Seq dataset of TBX3iso1 and TBX3iso2 transfectant cells [19]. Interestingly, the cell lines had distinct expression signatures and clustering patterns (Fig. 2a), suggesting differential transcriptional function of the TBX3 isoforms. We observed 470 differentially expressed genes between TBX3iso1 and TBX3iso2 overexpressing cell lines (FDR < 0.05). Pathway analysis of genes up-regulated with TBX3iso1 overexpression identified its potential role in OPN signaling, glycolysis/gluconeogenesis, and angiogenesis (Fig. 2b). Alternatively, pathway analysis of genes up-regulated with TBX3iso2 overexpression identified alterations in cytokines and inflammatory responses (Fig. 2c). Notably, TBX3iso2 overexpression resulted in down-regulation of genes involved in glycolysis/gluconeogenesis, showing inverse patterns from TBX3iso1 overexpressing cells.

Consistent with our finding of an enrichment of angiogenesis-related genes in response to TBX3iso1 overexpression, gross examinations of xenografts formed from TBX3iso1 overexpressing cells showed high tumor vascularity. To explore this phenomenon further, we assessed microvascular density by conducting immunohistochemical staining for the endothelial cell marker CD31. Microvascular quantification revealed significantly higher vessel density in TBX3iso1 tumors relative to the TBX3iso2 tumor for the majority (4/6) of tumors tested (Fig. 2d). In order to functionally examine for angiogenic properties, we collected conditioned media from 21NT transfectant cells and incubated them with HDMECs on Matrigel for 16 h to allow for endothelial cell migration and formation of tubule structures, which mimics the process of angiogenesis in vitro (Fig. 2e). We observed marked differences in promoting angiogenesis in vitro between conditioned media from the cell lines. Conditioned media from cells overexpressing TBX3iso1 promoted significantly higher rates of tubule formation by endothelial cells relative to conditioned media from TBX3iso2 overexpressing cells or the empty vector control.

### OPN is specifically up-regulated by TBX3iso1

**Fig. 3:**
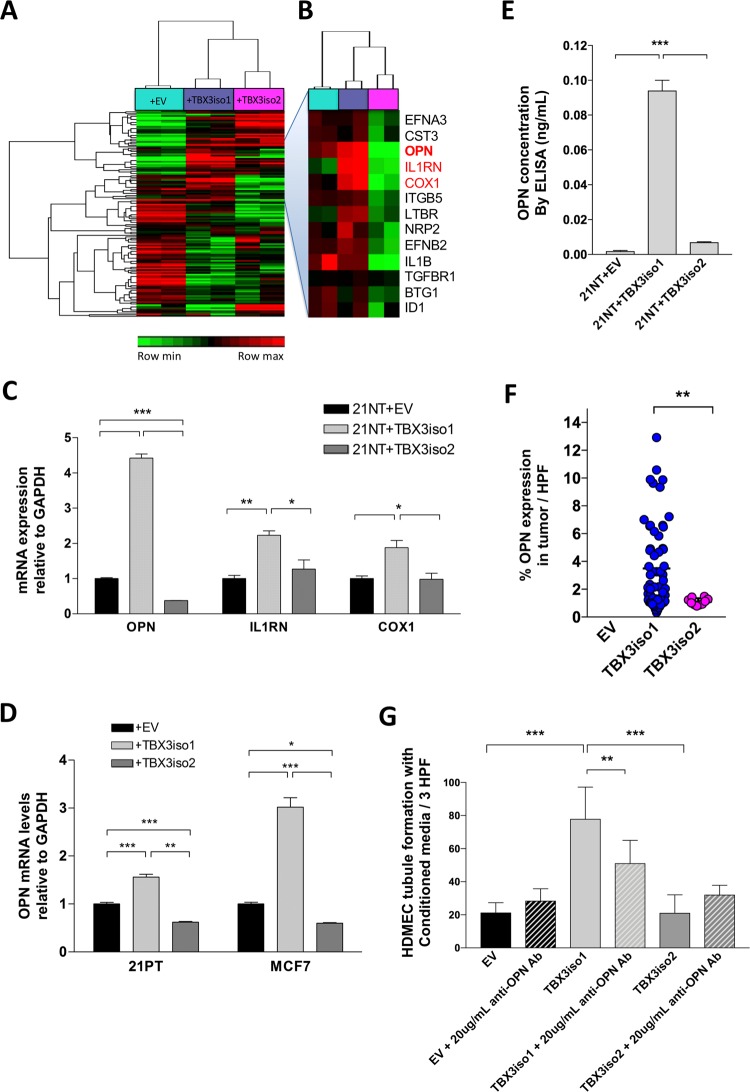
OPN is specifically up-regulated by TBX3iso1. **a** Heat map showing expression of 222 angiogenesis-related transcripts by RNA-Seq across 21NT + EV, 21NT + TBX3iso1, and 21NT + TBX3iso2 cell lines. **b** Cluster showing up-regulation of pro-angiogenic transcripts with TBX3iso1 overexpression and down-regulation with TBX3iso2 overexpression. Osteopontin (OPN), IL1RN, and COX1 are shown in red. **c** Expression of OPN, IL1RN, and COX2 mRNA levels in 21NT transfectant cell lines was assessed by qRT-PCR, normalized to GAPDH expression levels, and depicted as fold changes relative to the empty vector control. **d** Expression of OPN mRNA levels in 21PT and MCF7 transfectants was assessed by qRT-PCR, normalized to GAPDH levels, and depicted as fold changes relative to the empty vector control. **e** OPN protein levels in conditioned media was assessed by ELISA and normalized to cell numbers. Human recombinant OPN (hrOPN) was used to generate a standard curve for determination of absolute OPN protein concentration. **f** Primary tumors were stained for OPN by immunohistochemistry. Slides were scanned, digital images were thresholded and the percentage of positive signal within the image area was quantified. The percentage of the high power field (HPF) positive for OPN is shown across ten fields of view per tumor. **g** Tubule formation assay with OPN neutralizing antibody to assess in vitro angiogenesis. Conditioned media was collected after a 48 h incubation with 1.0 × 10^6^ cells of each cell type. Conditioned media was tested (either in the presence or absence of 20µg/mL anti-OPN antibody) by incubating with human dermal microvascular endothelial cells (HDMEC) on growth factor reduced Matrigel (96-well format) for 16 h at 37 °C to allow for vascular channel formation. The number of tubule branch points per three high-power fields (one well) was quantified at 16 h mark. *p < 0.05, **p < 0.01, ***p < 0.001 by one-way ANOVA with Tukey post-hoc for comparison between three groups, and student’s *t* test for comparison between two groups. Error bars represent standard deviation

In order to explore differences in transcriptional profiles between the two TBX3 isoforms with respect to angiogenesis-related genes, we generated an angiogenesis gene signature consisting of 222 genes using publicly-available databases (as described in Bioinformatics Analysis Methods, Table 2) and conducted hierarchical clustering of RNA-Seq data for all cell lines (Fig. 3a). Investigation of the depicted cluster at a higher resolution revealed up-regulation of a subset of pro-angiogenic transcripts in TBX3iso1 overexpressing cells and down-regulation in TBX3iso2 overexpressing cells relative to the empty vector (Fig. 3b). Genes showing the most pronounced differences between TBX3iso1 and TBX3iso2 transfectants (OPN, COX1, and IL1RN) were validated by qRT-PCR (Fig. 3c). Of note, and although unchanged with TBX3iso1 overexpression and therefore not within our list of genes, VEGFR2 was significantly down-regulated with TBX3iso2 overexpression. The VEGF family is commonly referred to as the main pro-angiogenic factors across cancer subtypes [28, 29], with VEGFR2 acting as the key receptor responsible for mediating VEGF-induced angiogenic activity [30]. For this reason, expression of VEGFR2 was also validated by qRT-PCR and found to be down-regulated in TBX3iso2 overexpressing cells (Supplementary Fig. 1).

We focused our further studies on OPN, since its mRNA levels displayed the greatest difference between TBX3iso1 and TBX3iso2 overexpressing cells, and OPN is known to be pro-angiogenic [30–34]. OPN expression was then further assessed in a series of stable TBX3 isoform transfectant cell lines (TBX3 expression levels for 21PT and MCF7 transfectant cell lines are shown in Supplementary Fig. 2a, b). Overexpression of TBX3iso1 resulted in significant up-regulation of OPN mRNA levels in all three (21NT, 21PT, and MCF7) transfectant cell lines (Fig. 3c, d). In addition, OPN mRNA levels were significantly down-regulated in all three cell lines overexpressing TBX3iso2. We then proceeded to evaluate the levels of OPN protein released into the conditioned media previously used for in vitro tubule formation assays. OPN protein levels were quantified by ELISA by comparing with recombinant human OPN standards (Fig. 3e). Relative to the empty vector control, there was a 49.0-fold increase in secreted OPN levels for TBX3iso1 overexpressing cells. As another mode of confirmation, we conducted immunohistochemical staining for OPN in the xenograft primary tumors (Fig. 3f). Consistent with our previously reported results, we observed a significantly higher proportion of OPN positive tumor cells in TBX3iso1 tumors relative to the single TBX3iso2 tumor, assessing across ten fields of view per sample. In order to assess whether the induction of OPN expression by TBX3iso1 was a significant contributing factor in the promotion of angiogenesis in vitro, we conducted tubule formation assays with the addition of an OPN neutralizing antibody (Fig. 3g). Importantly, we were able to significantly reduce vascular channel formation in vitro by blocking OPN, suggesting functional importance of OPN in TBX3-induced angiogenesis in vitro.

### TBX3iso1 overexpression leads to increased HAS2 levels and pericellular hyaluronan (HA) retention

**Fig. 4:**
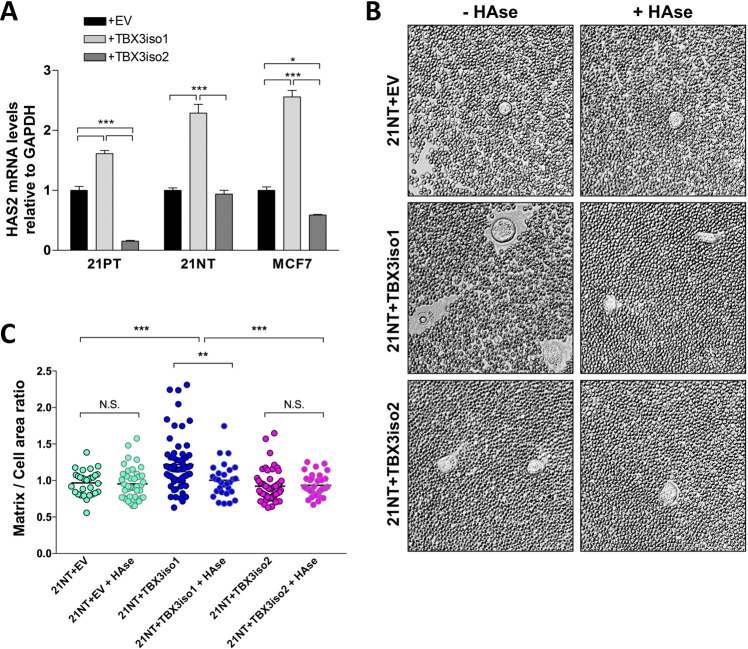
TBX3iso1 overexpression leads to increased HAS2 levels and pericellular hyaluronan retention. **a** Expression of HAS2 mRNA levels in 21PT, 21NT, and MCF7 transfectant cell lines was assessed by qRT-PCR, normalized to GAPDH expression levels, and depicted as fold changes relative to the empty vector control. **b**, **c** Particle exclusion experiment for visualization of pericellular matrices. Cells were pretreated in the presence or absence of hyaluronidase for 20 min, followed by removal of media and addition of fixed sheep erythrocytes. Pericellular matrix area was calculated by tracing around cell coats (matrices) and cell areas across 30 randomly selected cells. A ratio of 1.0 indicated the absence of a pericellular matrix for a particular cell. *p < 0.05, **p < 0.01, ***p < 0.001 by one-way ANOVA with Tukey post-hoc for comparison between three or more groups. Error bars represent standard deviation

We have previously shown that OPN induction of hyaluronan synthase 2 (HAS2) and hence HA production is associated with aggressiveness of 21NT cells [25]. Given that all three of the cell lines we tested (21PT, 21NT, MCF7) showed up-regulation of OPN only with TBX3iso1 overexpression, we proceeded to examine HAS2 mRNA levels. For all three cell line transfectants, overexpression of TBX3iso1 was associated with a significant up-regulation of HAS2 expression (Fig. 4a). Alternatively, overexpression of TBX3iso2 resulted in down-regulation of HAS2 mRNA expression in 21PT and MCF7 transfectant cell lines, with no change in HAS2 expression in the 21NT transfectant cell line. HA is produced by hyaluronan synthase enzymes at the intracellular face of the plasma membrane, and then either extruded from the cell and released into the microenvironment, or retained in pericellular coats [35–37]. High levels of synthesis and retention of HA in pericellular coats plays an important role in malignant progression [25], and is thus an indicator of poor prognosis in epithelial cancers [38–40]. In order to examine the phenomenon of HA retention, particle exclusion experiments were conducted using fixed sheep erythrocytes to visualize matrix production and retention (Fig. 4b, c). Due to the unique biochemical properties of HA, including its highly polar structure and large size, addition of partially negatively charged erythrocytes allows for visualization of matrices due to erythrocyte exclusion. Cells overexpressing TBX3iso1 displayed significantly larger pericellular coats relative to TBX3iso2 overexpressing cells and the empty vector control, suggesting higher levels of HA production. Administration of HAse completely abolished matrix assembly in TBX3iso1 overexpressing cells, confirming the presence of HA in the pericellular coats.

### Cancer progression involves an increase in the TBX3iso1 to TBX3iso2 ratio

**Fig. 5:**
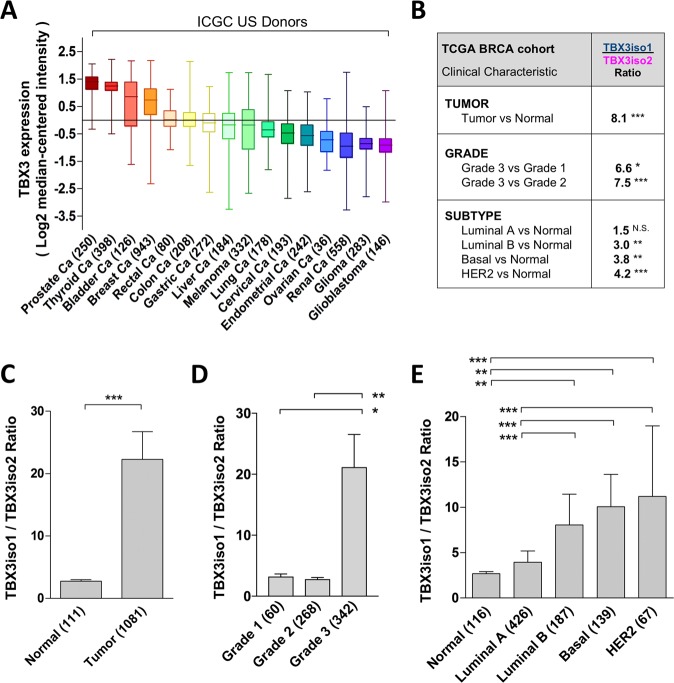
Cancer progression involves an increase in TBX3iso1 to TBX3iso2 ratio. **a** Assessment of the International Cancer Genome Consortium (ICGC) data shows total TBX3 mRNA levels across tumor subtypes (shown in various colors). Total TBX3 mRNA expression in breast cancer is shown in orange. **b** TBX3 isoform ratios were interrogated in the Cancer Genome Atlas (TCGA) breast cancer (BRCA) cohort by calculating the total transcript reads for TBX3iso1 over the total transcript reads for TBX3iso2 for each patient. Ratios were compared between normal and tumor tissues (**c**), and compared across tumor characteristics including grade (**d**) and molecular subtype (**e**). *p < 0.05, **p < 0.01, ***p < 0.001 by the nonparametric Kruskal–Wallis statistic with Dunn’s post-hoc test due to non-Gaussian distribution of ratios. Error bars represent standard deviation

TBX3 is overexpressed in several different cancer types [3–7, 9–12]. Examination of ICGC data revealed breast cancer (BRCA) as amongst the top TBX3 mRNA expressers across all tumor tissue sites (Fig. 5a). We then interrogated TBX3 isoform expression in the TCGA BRCA cohort, assessing the ratio of transcript reads for TBX3iso1 over TBX3iso2 for each patient and comparing with clinical characteristics (summarized in Fig. 5b). We report an 8.1-fold higher TBX3iso1 to TBX3iso2 ratio in tumor samples relative to control tissue (Fig. 5c). Importantly, this ratio was positively associated with higher tumor grade (Fig. 5d) and more aggressive breast cancer molecular subtypes (Fig. 5e). We then assessed TBX3iso1 to TBX3iso2 ratios in additional tumor types which overexpress TBX3, including melanoma [7], colon [8], and pancreatic cancer [9]. We observed an upwards shift in TBX3iso1 expression and downwards shift in TBX3iso2 expression within tumor tissues (TCGA dataset) relative to an expanded cohort of normal tissue controls (GTEx normal tissue dataset) (Supplementary Fig. 3), showing that this transcriptional shift is present across several cancer types.

## Discussion

Alternative splicing is a global post-transcriptional mechanism that adds an enhanced layer of complexity and diversification to genes encoded in the genome. Interestingly, alternative-splicing based transcriptional analyses and assessment of exon inclusion events have been shown to out-perform gene expression-based survival predictors across six different cancer types, including breast cancer [41]. Existing literature examining TBX3-induced tumorigenicity through the use of xenograft models [7, 42, 43], along with a single published transgenic TBX3-inducible mouse model [44], have often focused on only one isoform of TBX3 in different parental cell lines, which makes comparison of results between studies difficult. Liu et al. showed that inducible overexpression of TBX3iso2 in murine mammary glands results in mild focal hyperplasia and importantly no tumor formation [44]. For several other studies, which isoform was cloned and overexpressed is not stated. In addition, studies which employed knockdown of total TBX3 expression followed by xenotransplantation of cells into mice has revealed that TBX3 is associated with tumor formation, but not whether functional differences exist between isoforms. We therefore sought to investigate the functional consequence of this highly conserved splice event as it relates to TBX3-induced tumorigenesis. Through nude mouse xenograft experiments utilizing non-tumorigenic 21NT cell lines, we have reported significant differences between TBX3 isoforms in the promotion of tumorigenesis, with TBX3iso1 overexpression more commonly associated with initiation and progression of invasive carcinoma in vivo. This difference is likely associated with differing ability to induce angiogenesis, and is related to differential expression of a number of angiogenesis-associated genes.

While angiogenesis plays a limited role in normal adult physiology, it is a fundamental requirement in tumor growth [28, 45, 46]. After cytokines are released from cells, they are able to diffuse into the extracellular milieu and act on nearby quiescent endothelial cells to induce proliferation and migration towards the tumor [28]. As observed by in vitro tubule formation assays, pro-angiogenic factors are released from cells overexpressing TBX3iso1 that are functional in activating endothelial cells. We report that one key pro-angiogenic factor up-regulated with TBX3iso1 overexpression is OPN. Clinically, elevated OPN levels are associated with several breast cancer-related prognostic factors, including early metastasis and poor outcome [47–52]. OPN is described to act as a cytokine in various contexts [30, 53, 54], with several studies ascribing it a pro-angiogenic function [30–33]. Importantly, we found that addition of an OPN neutralizing antibody to conditioned media significantly reduced endothelial cell vascular channel formation, confirming OPN as a critical mediator of TBX3iso1-induced angiogenesis. We have previously reported OPN-induced up-regulation of HAS2 in 21NT breast cancer cell lines is necessary for both anchorage-independent growth and adhesion of tumor cells to bone marrow endothelial cells [25]. A great deal of overlap exists between the cellular functions affected by OPN and HA [55]; the two markers have both been correlated with cancer survival [38, 39, 47–51, 56] and are frequently co-expressed [55, 57]. Moreover, both HA and OPN are ligands for CD44 (in this case on endothelial cells) [58], which promotes angiogenesis through stimulation of endothelial cell migration, survival, and lumen formation [59–66].

Our novel in vivo findings relating to differential tumor-promoting effects between TBX3 isoforms and downstream confirmation of associated pathways suggests that the assessment of relative levels of splice variants may be more important than the assessment of total transcript levels per gene [67–69]. This focus on isoform ratios has been suggested by several studies [28, 67, 70, 71]. Through assessment of isoform ratios for breast cancer-related genes, Venables et al. reported cancer-specific exon loss for several transcripts, as well as a significant overlap with previously identified ovarian cancer-specific splice changes [72, 73]. This suggests that a subset of alternative splicing events may be common across cancer subtypes, which appears to hold true for alternative splicing of TBX3 as well.

It is unknown whether this increase in TBX3iso1 relative to TBX3iso2 expression is a cause or effect of the tumorigenic process; a splice shift phenomenon may be due to several factors, including potential epigenetic changes within the TBX3 gene, or due to changes in upstream splicing machinery [28, 74]. Recent studies have revealed that splice changes and isoform shifts in cancer are non-random and play a key role in cancer progression [28, 70, 71]. Based on these findings, various strategies are being employed in an attempt to exploit alternative splicing in diagnosis, prognosis, and treatment of cancer [28, 70, 75].

It is well established that TBX3 is aberrantly overexpressed in several cancer types [3–12]. The present study provides novel findings regarding the function of specific TBX3 isoforms, with elevated TBX3iso1 levels associated with several clinicopathological parameters, as well as a pro-angiogenic gene signature and angiogenesis-enhancing functions. It is therefore of importance to assess downstream transcriptional targets of both TBX3 isoforms in order to thoroughly understand transcriptional changes mediated by each isoform in disease states, including cancer.

## Supplementary information

Supplementary Figures
