# Supplementary material for: Isoform-specific promotion of breast cancer tumorigenicity by TBX3 involves induction of angiogenesis
Source: Lab Invest. 2019 Sep 30;100(3):400–13. doi: 10.1038/s41374-019-0326-6 (PMC7044113; doi:10.1038/s41374-019-0326-6)
Supplement: Supplementary file 1 — Supplementary Figures [file 41374_2019_326_MOESM1_ESM.pdf]

S1) Validation of relative VEGFR2 transcript levels by qRT-PCR.

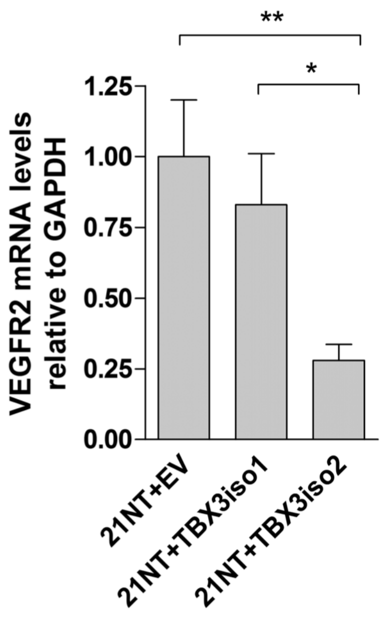

Supplementary Figure 1. Validation of relative VEGFR2 transcript levels by qRT-PCR.

VEGFR2 mRNA expression was assessed across 21NT transfectant cell lines by qRT-PCR, normalized to GAPDH expression levels, and depicted as fold changes relative to the empty vector control.

*p<0.05, **p<0.01, ***p<0.001 by one way ANOVA with Tukey post-hoc for comparison between three groups. Error bars represent standard deviation.

S2) TBX3 expression in stable cell line transfectants.

A

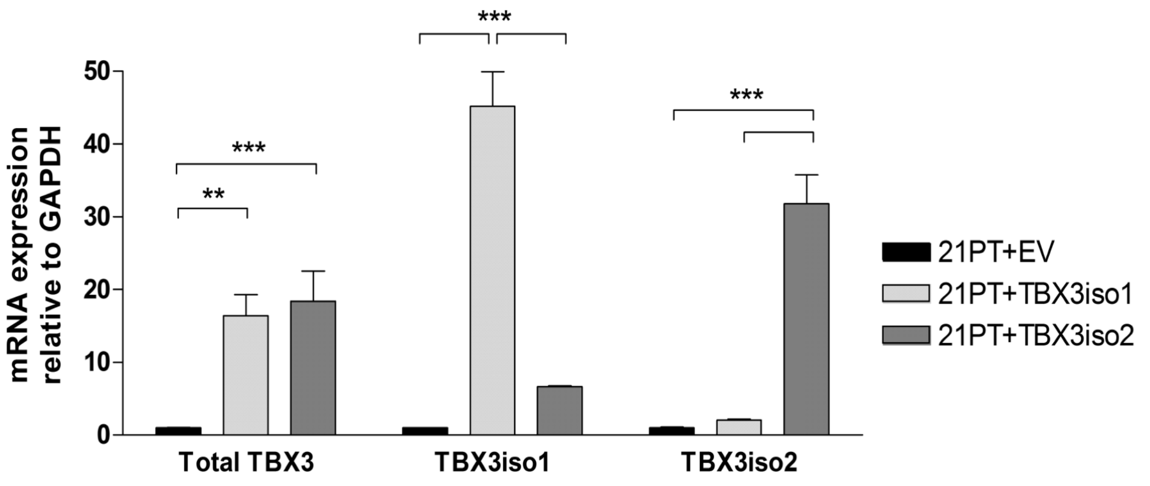

B

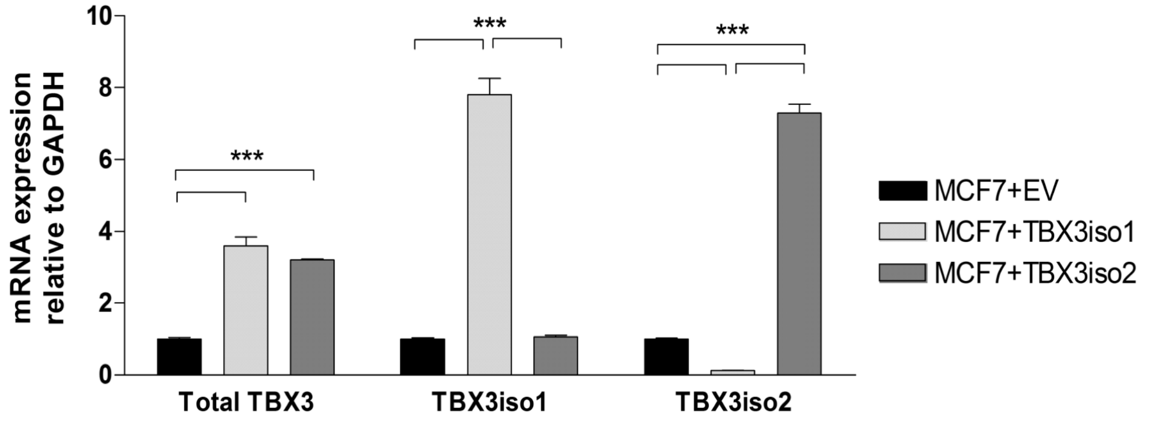

Supplementary Figure 2. TBX3 expression in stable cell line transfectants.

(A) Total TBX3, TBX3iso1, and TBX3iso2 mRNA expression was assessed by qRT-PCR in 21PT transfectant cell lines, normalized to GAPDH expression levels, and depicted as fold change relative to the empty vector control. (B) Total TBX3, TBX3iso1, and TBX3iso2 mRNA expression was assessed by qRT-PCR in MCF7 transfectant cell lines, normalized to GAPDH expression levels, and depicted as fold changes relative to the empty vector control.

*p<0.05, **p<0.01, ***p<0.001 by one way ANOVA with Tukey post-hoc for comparison between three groups. Error bars represent standard deviation.

S3) Assessment of TBX3 isoform expression in the TCGA and GTEx datasets.

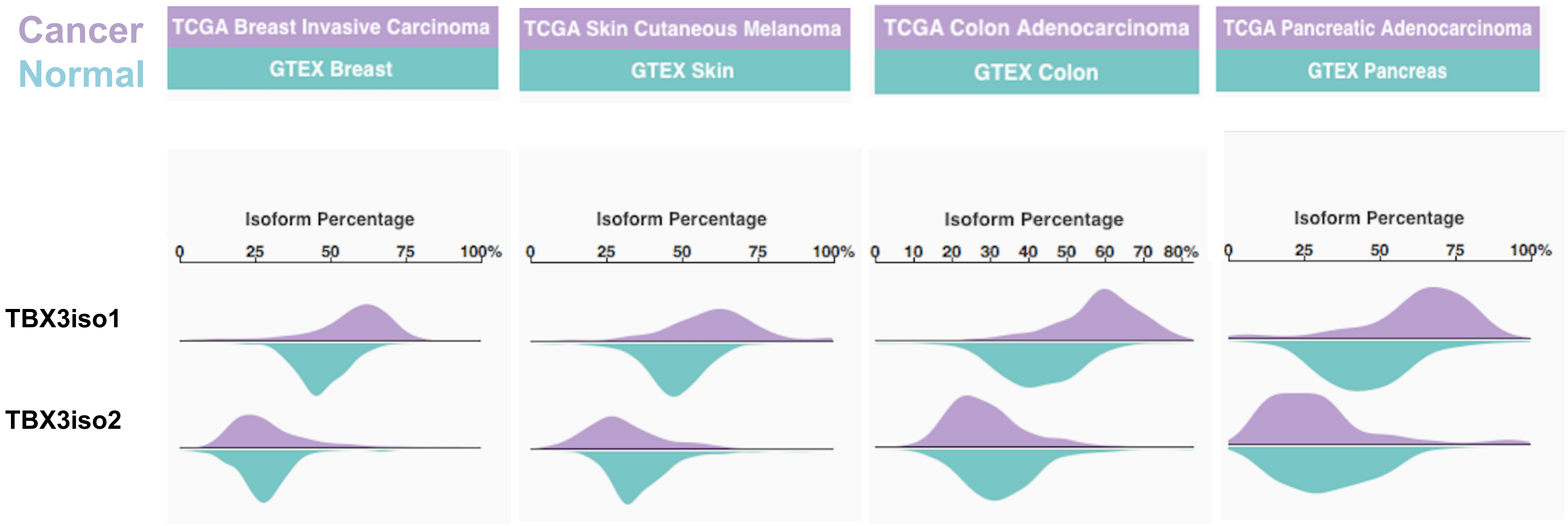

Supplementary Figure 3. Assessment of TBX3 isoform expression in the TCGA and GTEx datasets. TBX3 isoform transcript levels were assessed across tumor types in which TBX3 overexpression has been documented (breast cancer, melanoma, colon, and pancreatic cancer). Isoform percentage relative to all TBX3 transcripts is shown along the top axis, with TBX3 isoform percentage shown in tumors (TCGA; purple hills) and normal tissues (Genotype Tissue Expression Project, GTEx; blue hills). Data was assessed using XenaBrowser.
